# Understanding the Roles of Host Defense Peptides in Immune Modulation: From Antimicrobial Action to Potential as Adjuvants

**DOI:** 10.4014/jmb.2301.01005

**Published:** 2023-02-10

**Authors:** Ju Kim, Byeol-Hee Cho, Yong-Suk Jang

**Affiliations:** 1 Department of Molecular Biology and the Institute for Molecular Biology and Genetics, Jeonbuk National University, Jeonju 54896, Republic of Korea; 2 Innovative Research and Education Center for Integrated Bioactive Materials and the Department of Bioactive Material Science, Jeonbuk National University, Jeonju 54896, Republic of Korea; 3 Department of Bioactive Material Sciences and Research Center of Bioactive Materials, Jeonbuk National University, Jeonju 54896, Republic of Korea

**Keywords:** Adjuvant, antimicrobial peptide, immune response, infection, vaccine

## Abstract

Host defense peptides are expressed in various immune cells, including phagocytic cells and epithelial cells. These peptides selectively alter innate immune pathways in response to infections by pathogens, such as bacteria, fungi, and viruses, and modify the subsequent adaptive immune environment. Consequently, they play a wide range of roles in both innate and adaptive immune responses. These peptides are of increasing importance due to their broad-spectrum antimicrobial activity and their functions as mediators linking innate and adaptive immune responses. This review focuses on the pleiotropic biological functions and related mechanisms of action of human host defense peptides and discusses their potential clinical applications.

## Introduction

Host defense peptides (HDPs), also known as antimicrobial peptides (AMPs), have been characterized in all living organisms, including microorganisms, plants, animals, and humans [1]. In humans, HDPs show a broad range of antimicrobial activities and play roles in immune activation, including a primary role in innate immunity [2]. A large number of HDPs have been identified to date, consisting of more than 2,600 natural AMPs and a number of immunomodulatory peptides [3]. They are generally small peptides (30–60 amino acids) characterized as strong cations, which are heat-stable and have no effect on eukaryotic cells. An online database of HDPs (http://aps.unmc.edu/AP/main.php) categorizes these molecules based on their three-dimensional secondary structures: the α family, which includes an α-helical structure; the β family characterized by the presence of at least a pair of β-strands; the αβ family, which contains both α-helical and β-strand structures; and the non-αβ family, which has neither α-helical nor β-strands [4, 5]. A total of 147 human HDPs have been annotated to date, along with 352 in mammals, 1,148 in amphibians, 140 in fish, 45 in reptiles, 43 in birds, and 600 in arthropods.

**Fig. 1:**
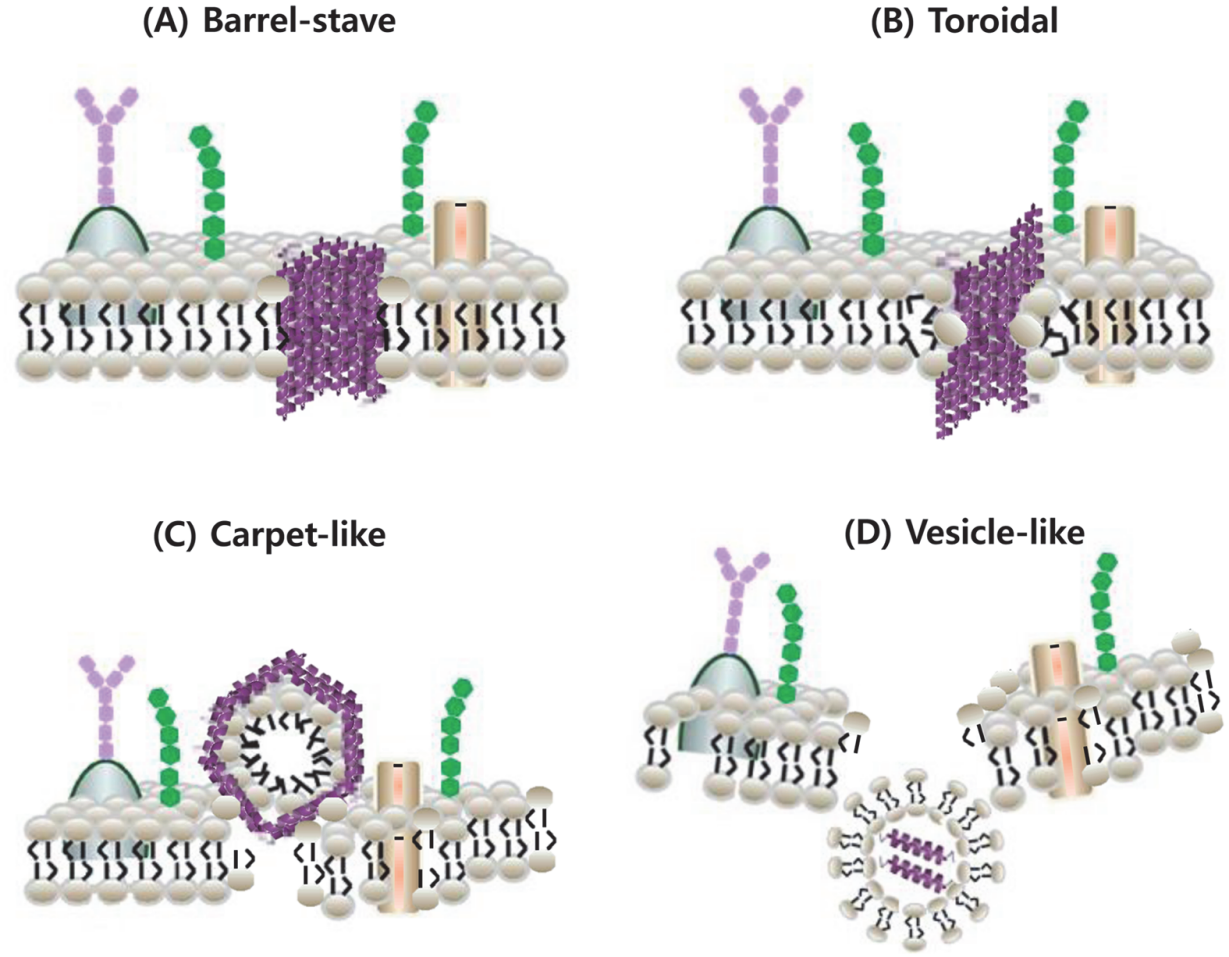
Modes of action of HDPs. HDPs appear to function either through pore formation by direct interaction with the cell membrane, or within cells after escape from vesicle-like bodies. Modes of pore formation include barrel-stave (**A**), toroidal (**B**), and carpet-like (**C**) models, while the vesicle-like body model involves endocytosis-based translocation (**D**). (**A**) In the barrelstave model, HDPs bind the target cell membrane and are inserted into the hydrophobic lipid core of the plasma membrane to form barrels. Thereafter, the cytoplasmic contents are leaked through transmembrane pores, resulting in cell lysis. (**B**) In the toroidal model, HDPs are inserted into the target cell membrane and the head group of the lipid monolayer aligned with HDPs is bent, forming transmembrane pores and resulting in cell death in conjunction with depolarization of the plasma membrane. (**C**) In the carpet-like model, HDPs cover the surface of the target cell membrane, destroying the plasma membrane and forming carpet-like micelles. The interaction of the cell membrane and HDPs leads to the formation of pores in the inner membrane, followed by cell lysis. (**D**) The vesicle-like bodies model is associated with endocytosis for uptake of large particles. Vesicle-like bodies, such as macropinosomes formed by inward folding of the outer membrane, contain HDPs and translocate into the cell. After endosomal escape for intracellular delivery of HDPs, these peptides act on intracellular targets.

The emergence of antibiotic-resistant microorganisms has become a worldwide public health issue [6], which has prompted increasing interest in the pharmacological application of HDPs. The basic antimicrobial mechanism of action of HDPs is via electrostatic interaction with negatively charged molecules on the microbial membrane [7]. In addition, antimicrobial activity of HDPs can be exerted through cell membrane translocation and inhibition of essential cellular processes, such as synthesis of nucleic acids, cell wall components, and proteins in target cells [8]. The mode of action of HDPs can also be used to categorize them into membrane acting peptides, such as defensin and LL-37, and nonmembrane-acting peptides, such as human neutrophil peptide (hNP)-1, pleurocidin, and dermaseptin [9]. Membrane damage in target cells induced by HDPs is mediated by pore formation, thinning of the membrane, and/or disruption of the lipid bilayer, as summarized in Fig. 1 [10, 11].

HDPs show antimicrobial specificity, destroying specific target cells without affecting host cells. In addition to their antimicrobial activity, HDPs have multiple functions, including immunomodulatory activities, and play a primary role in linking innate and adaptive immune responses [12]. They have also been reported to function as antitumor agents, drug delivery systems, and signaling molecules in the immune system. For example, LL-37, which exhibits antimicrobial and antiviral activity, can be applied to prevent infection by *Pseudomonas aeruginosa*, influenza viruses, and respiratory syncytial virus in vivo [13]. LL-37 has also been shown to act in a protective mode that involves an early enhanced neutrophil response rather than direct microbicidal activity [14]. Moreover, β-defensin 2 has been shown to contribute to antitumor natural killer and beneficial T-cell responses via dendritic cell (DC) activation in murine models in vivo [15]. These results suggested that HDPs in the complexed form with antigens may have adjuvant potential for use in clinical applications, because the mechanisms of immunomodulatory action of HDPs are fundamentally different from those of conventional adjuvants [16]. However, studies regarding whether HDPs can be applied as prophylactic vaccines or therapeutic agents against infectious diseases are in the early stages. This review discusses our current understanding of the functions of HDPs, including their potential application as antimicrobial agents and immunoregulators.

## Major Types of HDPs

HDPs are expressed in a wide range of tissues and cell types in various species, including mammals, amphibians, and fish. In humans, most of these peptides are synthesized in epithelial layers or are secreted by circulating immune cells, including neutrophils and tissue mast cells [17]. HDPs have diverse sequences, unique structures, and target specificity. Among the HDPs, defensins are small cysteine-rich cationic proteins typically composed of 18–45 amino acids, with three to four highly conserved disulfide bonds. They show direct antimicrobial and/or immune signaling activities against bacteria, fungi, and enveloped and nonenveloped viruses. The major families of human HDPs that are of interest from the perspective of clinical application are summarized below.

Human defensins are classified as α-defensins (hNPs) and β-defensins (hBDs) based on the positions of cysteine residues, peptide chain folding, and length. The respective bioactivities of these peptides depend on the localization of their expression according to the state of health and/or infection of hosts [18]. Six types of hNPs have been identified to date: hNP-1, hNP-2, hNP-3, hNP-4, hNP-5, and hNP-6. hNPs, which are mainly secreted by neutrophils, have almost identical amino acid sequences but differ in their N-termini, which appear to change the antimicrobial spectrum. For example, hNP-1 and hNP-2 are active against *Staphylococcus aureus*, *P. aeruginosa*, and *Escherichia coli*, whereas hNP-3 and hNP-4 actively destroy *Candida albicans*, *E. coli*, and *Streptococcus faecalis* [19]. Both hNP-5 and hNP-6 are expressed in the gastrointestinal tract, but not in the oral cavity [20]. The concentration of hNP-1 is high in patients with leukoplakia and squamous cell carcinoma [21]. Patients with dental caries have low levels of hNP-1, hNP-2, and hNP-3, which are used as indicators of caries risk [22]. The β-defensins (BDs) are small cationic peptides with antimicrobial activity that are mainly expressed in epithelial cells of various tissues, including the skin, oral cavity, gastrointestinal tract, respiratory tract, and kidneys [23-25]. Various hBD expression patterns have been reported in relation to their locations and status according to stage of infection. Of the 28 hBDs identified to date, only hBD-1 to hBD-4 have been detected in the oral epithelium [26]. hBD-1 and hBD-2 are localized in the oral suprabasal layer, and hBD-3 is expressed in the basal layer of undifferentiated epithelial cells [3]. hBD-1 is expressed continuously to prevent normal flora from becoming opportunistic, while hBD-2 and hBD-3 are more protective against pathogens and are induced in response to proinflammatory mediators, such as lipopolysaccharide (LPS), tumor necrosis factor (TNF)-α, interleukin (IL)-1β, and interferons (IFNs) [27].

The human cathelicidin HDP, LL-37, does not have cysteine residues and belongs to the amphiphilic α-helical peptide family. Human cationic AMP 18 (hCAP18), a propeptide of LL-37, consists of a highly conserved cathepsin-L inhibitor (cathelin)-like domain and a C-terminal peptide [28]. hCAP18/LL-37 is the only cathelicidin found to date in neutrophils, monocytes, and epithelial cells of the respiratory tract and oral cavity [29, 30]. LL-37 has been reported to bind and neutralize the activity of bacterial LPS and to inhibit the reverse transcriptase activity of human immunodeficiency virus (HIV)-1 [31]. LL-37 has potent antimicrobial activity against many bacteria, fungi, viruses, and parasites [32]. In addition, LL-37 acts as a chemotactic factor for various immune cells, including DCs, macrophages, neutrophils, mast cells, and T cells [33].

HDPs have been referred to as natural antibiotics, which have multifunctional properties with a broad spectrum of bioactivity against bacteria, fungi, viruses, parasites, and tumor cells, and therefore have potential as therapeutic agents. In general, the positively charged HDPs interact directly with the negatively charged cell membranes of target cells to increase membrane permeability, resulting in rapid cell death [34]. In addition, most HDPs have been reported to function in recruitment, activation, and maturation of inflammatory and immune cells or tissue repair as part of the primary host responses to pathogen invasion [17]. The major biofunctions of HDPs can be classified as antibacterial, antifungal, antiviral, and immunomodulatory activities. The functions of HDPs and underlying mechanisms of action are described in detail in the following sections.

## Antibacterial Activity

HDPs, especially those isolated and characterized from higher organisms, exhibit a broad spectrum of bioactivity against microbial pathogens, including Gram-positive and Gram-negative bacteria [8], fungi [35], and viruses [36]. The mechanisms underlying the antibacterial actions of HDPs appear to vary depending on microbial pathogens, and the antimicrobial effects of HDPs on a broad range of pathogens have been used as the basis for the development of broad-spectrum antimicrobial agents. HDPs with antibacterial activity against Gram-positive bacteria are relatively rare compared to those with other types of antimicrobial activity. Isoform 5, which was isolated from the hemolymph of immunized Udo longicorn beetle (*Acalolepta luxuriosa*) larvae, is a representative peptide with antimicrobial activity against the Gram-positive bacterium *Micrococcus luteus* [37]. Many HDPs with activity against Gram-negative bacteria have been identified compared to those with activity against Gram-positive bacteria. Hinnavin II, a typical peptide that is more effective against Gram-negative than Gram-positive bacteria, showed strong synergistic effects with purified lysozyme to inhibit bacterial growth [38]. As there is extensive literature on the antibacterial actions of HDPs, only a few illustrative examples of the biological activities of human defensins are listed below.

Giesemann *et al*. reported that hNP-1, hNP-3, and hNP-5 blocked toxin B, one of the main virulence factors of *Clostridioides difficile*, which has been implicated in antibiotic-associated diarrhea and pseudomembranous colitis [39]. These results suggest that human α-defensins can interact with toxin B to inhibit this toxin-induced glucosylation of Rho GTPases, and therefore represent a unique defense mechanism against bacterial cytotoxins [39]. Among the hBDs, hBD-2 showed strong bactericidal activity against Gram-negative bacteria and yeast, and exhibited bacteriostatic activity against Gram-positive bacteria and *S. aureus* [40]. In addition, the broad-spectrum antimicrobial activity of hBD-3 against Gram-negative and Gram-positive bacteria has also been investigated [41, 42]. hBD-3 was shown to have potent antimicrobial activity against multidrug-resistant clinical isolates of *S. aureus*, *Enterococcus faecium*, *P. aeruginosa*, *Stenotrophomonas maltophilia*, and *Acinetobacter baumannii* [43]. In addition, hBD-3 acts as an effective inhibitor of biofilm formation by methicillin-resistant *Staphylococcus epidermidis* in addition to methicillin-resistant *S. aureus* [44]. Other hBDs have also been shown to have antimicrobial efficacy. For example, hBD-5 and hBD-6 at high concentrations were shown to eliminate *E. coli* K12, but were inactive against *S. aureus* [45]. In addition, analyses of the antimicrobial activities of recombinant hBDs against *E. coli*, *S. aureus*, *P. aeruginosa*, and multidrug-resistant strains suggested that recombinant hBDs can be used against pathogens, including antibiotic-resistant/tolerant strains [46].

## Antifungal Activity

More than 70,000 species of fungi have been identified to date, and fungal infections by species such as *Aspergillus fumigatus*, *Cryptococcus neoformans*, *C. albicans*, and *Histoplasma capsulatum* are responsible for 1.5 million deaths per year worldwide [47]. Although the incidences of life-threatening fungal infections in humans are increasing worldwide, only limited antifungal agents are available for the treatment of severe invasive fungal disease [48]. Furthermore, the increasing number of immunocompromised patients worldwide is leading to higher rates of fungal infections, requiring the development of powerful new antifungal agents to which resistance does not readily emerge. Many HDPs show antifungal activity in addition to antibacterial activity. Antifungal HDPs have been found mainly in plants and vertebrates [49], and defensins from plants have been mainly studied in the context of antifungal activity [50]. Histatin 5, produced only in humans and higher primates, is a salivary cation histidine-rich peptide, which has been shown to affect mitochondrial function in *C. albicans* [51]. It was also reported that the membrane properties of *C. albicans* were affected by cathelicidin and HDP mimic compounds [52]. Among the venom peptides (OdVP-1, OdVP-2, and OdVP-3) isolated from the solitary wasp, *Orancistrocerus drewseni*, OdVP-2 and its analogue (OdVP-2L) showed strong antifungal activity, but OdVP-2L did not exhibit antibacterial activity or/and antimicrobial activity against yeasts [53]. Analysis of the minimum growth inhibitory concentration of Bactroserin-1 characterized from immunized *Bactrocera dorsalis* showed broad-spectrum antimicrobial activity against fungi and Gram-positive/negative bacteria [54]. The antimicrobial mechanisms of action of various HDPs against fungi and yeasts have been reported, and their diverse modes of action suggest that HDPs and their derivatives may be used in the development of potent antifungal agents.

## Antiviral Activity

Viruses infect and replicate within the cells of various organisms. However, some HDPs can eliminate viruses, and some peptides have also been reported to show inhibitory activity against certain viruses [55]. It was initially proposed that HDPs target enveloped viruses and directly damage viral membranes. Recently, however, some antiviral HDPs have been reported to act at multiple stages of the viral lifecycle, including host cell entry and viral replication [56]. Scorpine (Scp) isolated from the venom of the scorpion, *Pandinus imperator*, was found to inhibit viral replication of dengue virus serotype 2 in C6/36 mosquito cells [57]. The observed functions of β-defensin in plasmacytoid DCs (pDCs), one of the major classes of immune cells, supports their multifunctionality in the innate immune defense system against virus infection [58]. Ryan *et al*. also showed that PR8, HSV-1, and Sendai virus increased the gene expression and production of hBD-1 peptide in pDCs and suppressed the gene expression of hBD-1 in epithelial cells. Furthermore, their studies with HSV-1 showed that replication of HSV-1 occurred in epithelial cells but not in pDCs. Together, these results suggest that hBD-1 may play a role in preventing viral replication in pDCs [59]. In addition, there are studies that hBD-3 activates professional APC such as monocytes and dendritic cells in a TLR-dependent manner [60]. They showed that hBD-3 induces the expression of the costimulatory molecules including CD40, CD80, and CD86 on monocytes and DCs via TLR1 and 2. It is thought that hBDs possibly cause the maturation of DCs and the induction of proinflammatory cytokines by professional APCs, leading to more enhanced antigen presentation and subsequent T cell activation. Most studies on the antiviral activity of human defensins have focused on their potential as antiviral drugs against HIV, especially HIV-1 [61]. In addition to HIV, defensins have also been shown to be active against adenoviruses, cytomegaloviruses, enteroviruses, herpesviruses, influenza viruses, and other viruses (Table 1).

The antiviral mechanisms of action of HDPs are not completely understood. The unique characteristics of defensins, such as their cationic nature, amphiphilicity, and high hydrophobicity are considered to play roles in their antiviral effects. In addition, the diversity of virus species sensitive to defensins indicates that the antiviral mechanisms of action of defensins are not simple but mixed [62]. These mechanisms include a mode in which defensins directly target viral surface proteins, such as envelope and capsid proteins, as well as defensin-based inhibition of viral fusion and post-entry intracellular neutralization. In addition, defensins can bind receptors on the surface of the host cell, disrupting intracellular signaling pathways or inhibiting viral replication. Furthermore, the enhanced and/or altered adaptive immune responses to viruses by defensins suggest that defensins can act as efficient immunomodulators linking innate and adaptive immunity against virus infection [62].

## Immunomodulatory Actions of HDPs

Promotion of the innate immune system by HDPs has been suggested as a major mechanism for the early elimination of infectious agents. Most human HDPs are produced by epithelial, inflammatory, and immune cells as part of the host defense response to microbial invasion [17]. Early studies of the nonmicrobicidal properties of HDPs were mainly concerned with their effects on immune cells, such as the ability to recruit leukocytes [33, 63]. Subsequent studies revealed the various immunoregulatory functions of HDPs. This review focuses mainly on human defensins and cathelicidins to discuss the activities of these peptides on modulation of inflammatory and immune responses.

Several studies have shown that HDPs, including cathelicidins, have potent antiendotoxin properties in vitro and in vivo by binding bacterial LPS [64, 65] or by intervening in Toll-like receptor (TLR) signaling pathways [66]. For example, downregulation of HDP-mediated TLR to NF-κB signaling pathways in the inflammatory state results in the inhibition of certain proinflammatory responses, such as the production of TNFs and reactive oxygen species (ROS) [67, 68]. However, the modulation of TLR-mediated signaling pathways by HDPs is not necessarily anti-inflammatory because these peptides inhibit LPS-induced TNF production, while also inducing the production of chemokines, such as IL-8 and MCP-1, which may attract leukocytes [69]. Especially, cathelicidins exhibit pro- and anti-inflammatory activities, depending on the routes and stages of infection. In situations where the bacteria have been removed, LL-37 acts as an anti-inflammatory activator and prevents inflammatory activation via TLR2 and TLR4 [70]. In addition, LL-37 suppresses the production of proinflammatory cytokines, such as TNF, IL-6, and IL-1β, induced by IL-32 through activation of the bispecific phosphatase MKP1, a negative regulator of inflammation, without altering the induction of chemokine production [71]. Furthermore, LL-37 modulates cytokine-mediated immune responses in a variety of cell types. For example, LL-37 can induce the expression of members of the IL-1 family, including Th1 and Th17 cell-related cytokine genes, such as IL-6 and IL-23A, in keratinocytes [72]. Similarly, defensins including hBD-3 not only exhibit antiendotoxin properties in vitro and in vivo through modulation of TLR-mediated signaling pathways [73, 74], but also lead to the production of proinflammatory cytokines in monocytes and the maturation of DCs. In addition, hBD-3 has been reported to mediate in vivo adjuvant properties of noninflammatory DNA molecules [75]. These results demonstrate the potential of HDPs to modulate innate and adaptive immune responses through their impacts on signaling pathways of pattern recognition receptors and their crosstalk.

Furthermore, several studies have demonstrated the anti-inflammatory functions of HDPs by showing increased inflammatory responses when these peptides are deficient. Cathelicidin-deficient mice showed more severe inflammatory responses than wild-type controls [76], and decreased α-defensin expression in human enterocytes was shown to be associated with the development of Crohn’s disease [77]. In particular, exogenous application of HDPs, such as LL-37 and HBD-2, has been reported to control inflammation in diverse infectious and septic animal models [78-80]. In addition, LL-37 derivatives modulated disease progression in an animal model of inflammatory arthritis [81]. Animal studies in a septic cathelicidin-deficient mouse model showed increased survival rate despite increased proinflammatory gene expression [76]. Therefore, the outcome of HDP-mediated regulation of inflammatory responses is context-dependent and appears to be dependent on the cellular environment. As described above, HDPs exhibit multifunctional properties that activate proinflammatory responses to aid in the elimination of pathogens. In addition, potent HDP-mediated anti-inflammatory activities have also been observed, suggesting that HDPs are regulatory molecules that limit excessive inflammation. Therefore, HDPs are thought to act as regulators that can balance inflammation and anti-inflammatory responses to promote immune homeostasis.

Neutrophils are the major source of defensins and cathelicidins, and the primary innate immune effector cells that respond to the early stages of infection. HDPs promote the influx of neutrophils by direct chemotactic effects [82] and by inducing the secretion of chemokines that attract neutrophils, such as IL-8, in a mitogen-activated protein (MAP) kinase-dependent manner [83]. However, the functions of HDPs in modulating host cell responses to infections are not limited to their effects on neutrophils, but they also modulate other innate and adaptive cellular immune responses [84]. For example, immune cells, such as macrophages, mast cells, and T cells, exhibit direct chemotaxis toward HDPs and their derivatives [33, 85, 86]. In addition, HDPs indirectly lead to the recruitment of leukocytes by promoting the release of chemokines [87, 88]. These abilities of HDPs to induce the production and release of chemokines and promote the recruitment of leukocytes and immune cells have been considered primary immunomodulatory mechanisms associated with protection against infection [85, 89]. Their underlying molecular mechanisms involve several different cellular receptors, including chemokine receptors, such as CCR6 and CCR2, G protein-coupled receptors (GPCRs), such as the formyl peptide receptors [90], and TLRs [91], as well as selective interactions with intracellular proteins, such as p62 and GAPDH [92, 93].

As discussed above, HDPs appear to act as linkers between innate and adaptive immunity due to their abilities to recruit antigen-presenting cells (APCs), such as DCs, and macrophages, to sites of infection. In addition, HDPs can modulate the adaptive immune response by influencing the generation and polarization of immune cells, as well as by activating APCs. For example, defensins, such as hBD-2 and hBD-3, have been shown to induce the production of IFN-α in pDCs and consequently influence the initiation and magnitude of T-cell responses [94]. In addition, the levels of expression of M1-type macrophage marker genes, such as CD86 and CD16, on macrophage-like THP-1 cells are upregulated by treatment with hBD-2, enhancing the adaptive immune response through the promotion of CCR2-mediated Nod2 signaling [95]. In addition, hBDs have been reported to chemoattract and activate immature DCs and memory T cells [96]. Recruitment of DCs by hBDs is thought to facilitate the uptake, processing, and presentation of antigens by APCs, leading to activation of a broad and durable immune response. Therefore, hBDs appear to play major roles in both innate and antigen-specific adaptive immunity in the host [97]. The influence of HDPs on adaptive immunity has been studied mainly by the application of cathelicidin and defensin as adjuvants to enhance systemic and mucosal antigen-specific immune responses [94, 98, 99].

## Functional Mechanisms of Action of HDPs

HDPs possess similar physical properties that are responsible for their multifunctional activities. The potent antimicrobial activities of HDPs due to their cationic charge are related to the presence of multiple lysine, tryptophan, and arginine residues, and hydrophobicity or amphipathicity. The mechanisms of broad-spectrum antimicrobial activities of HDPs vary from cell membrane permeabilization to effects on intracellular molecules with immunomodulatory activity. HDPs can lead to cell lysis through membrane-destructive mechanisms, or they can lead to the transient formation of membrane pores and transport of these peptides into cells, eliciting selective responses via binding to intracellular targets [100]. As noted above, HDP-mediated microbicidal mechanisms are generally mediated through membrane permeabilization, but non-membrane-disruptive HDPs have also been reported [101]. These non-membrane-disruptive peptides are known to affect different internal cellular processes, including synthesis of macromolecules, such as DNA, RNA, and proteins [102]. In addition to their ability to interact with membranes, HDPs have been shown to interact with different target molecules within cells.

Structurally diverse cationic amphiphilic HDPs can show direct antimicrobial activity [103, 104]. Under the same conditions, HDPs exhibit extensive immunomodulatory activities, including the selective modulation of inflammatory and innate/adaptive immune responses, wound healing, and adjuvant-like responses that skew and enhance adaptive immune responses [105]. For example, macrophages, well known as APCs, are polarized into M1 and M2 macrophages, which promote proinflammatory and anti-inflammatory responses, respectively. Both M1 and M2 macrophages were shown to decrease TNF-α production in response to LL-37 [106], while LL-37 has also been shown to make M2 macrophages more proinflammatory [107]. Although cathelicidins influence APCs and the interaction of adaptive immune cells with APCs, cathelicidins have been shown to exert a more direct effect on adaptive immune responses. Mice immunized with mouse cathelin-related antimicrobial peptide (mCRAMP) and ovalbumin (OVA) showed increased OVA-specific IgG production compared to mice immunized with OVA alone [98]. Similarly, An *et al*. reported that LL-37 functions as an effective adjuvant for anticancer vaccines [108], and Davidson *et al*. reported that LL-37 regulates DC-induced T-cell polarization toward a Th1 response [109]. Recently, the direction and extent of the effects of APCs and innate immunity in these responses are increasingly being elucidated, although the relations between HDPs and adaptive responses are still under investigation.

The molecular mechanisms by which HDPs modulate immune responses in relation to pathogen clearance and immune homeostasis are highly complex [88, 110]. For example, intracellular uptake of HDPs may or may not be mediated by membrane-associated GPCRs. There are also interactions with intracellular proteins or receptors, such as GAPDH and p62, and alterations of several signaling pathways associated with NF-κB, p38, JNK MAP kinase, phosphoinositide 3-kinase, and other transcription factors. All of these phenomena appear to depend on the peptide concentration, reaction rate, and environmental stimuli.

## Applications and Future Prospects for Clinical Use of HDPs

HDPs, which were initially thought to exhibit only antimicrobial activity, have been shown to exert antiviral and immunomodulatory effects. HDPs are promising therapeutic agents due to their relatively low toxicity and reduced risk of tolerance in vivo. In addition, HDPs can be applied via a variety of routes through injection, oral administration, inhalation, and topical application. Accordingly, the anti-infective and therapeutic potentials of natural and synthetic HDPs are attracting increasing interest in both the pharmaceutical industry and academia.

Treatment of respiratory infections with HDPs, such as defensins and LL-37, appears to provide lung protection. For example, nebulizing LL-37 into mice prior to infection with influenza A virus was reported to reduce the severity of infectious disease and increase survival rate [111]. The same concept may also be applicable to the prevention and treatment of disease caused by other respiratory pathogens, including the use of HDPs, such as lactoferrin, which was previously shown to be effective against respiratory syncytial virus infection [112]. In addition, HDPs that directly target a variety of infectious viruses are being considered as alternatives to antiviral drugs associated with resistance.

In addition to antimicrobial functions, many studies have attempted to exploit the therapeutic potential of HDPs to modulate both innate and adaptive immune responses. LL-37 was the first HDP to be applied to a human randomized placebo-controlled trial, where it was shown to improve healing of venous leg ulcers without local or systemic side effects [113]. In addition, the application of immunomodulatory HDPs to wound healing is based on studies showing that growth factors associated with tissue regeneration induce the production of endogenous HDPs, such as LL-37 and β-defensin, in keratinocytes [114]. Due to the ability of HDPs to modulate inflammatory responses, many studies have explored the influence of these peptides on cancers. Application of hBD-2 was shown to enhance antitumor effects in preclinical anticancer studies using CT26, LL/2, and MethA cells. In addition, the mechanism of action of this peptide was shown to be related to its immunomodulatory ability with activation of endogenous DCs [115]. However, HDP-based therapy for cancer is controversial because the effectiveness of these peptides appears to be selective for the type of cancer [116].

HDPs, such as β-defensin and LL-37, can be used to improve vaccine platforms as adjuvants with a broad range of bioactivities [117]. Mei *et al*. showed that murine BD-2 (mBD-2) promotes antitumor responses in vivo, including the infiltration of T cells, NK cells, and macrophages into tumor tissues [15]. In addition, mBD-2, an endogenous ligand for TLR-4, induces DC maturation and upregulation of costimulatory molecules [96], and is thought to contribute to the establishment of Th1 responses that can link innate to adaptive immune responses by promoting the expression of cytokines, such as IL-12, IL-6, and IFN-γ [118]. Furthermore, hBD-2 and hBD-3 can promote the uptake of DNA molecules and the production of IFN-α in pDCs, consequently enhancing vaccine responses [94]. BDs act as chemoattractants for immune cells and are known to have significant structural similarity to chemokines. BDs appear to attract myeloid and lymphoid cells into the mucosal immune-inductive sites through CCR2 and CCR6 receptors, thereby linking innate and adaptive immunity [119]. Defensins have also been shown to affect B-cell responses [120], and are therefore involved in the maintenance of long-term cellular and humoral immune responses to pathogens [121], which are considered to be important factors in vaccine development.

In general, protein-based subunit vaccines are known to be less effective in inducing cellular immune responses, especially cytotoxic T lymphocyte activation, than nucleic acid or live attenuated vaccines. Recently, the possibility of antigen cross-presentation by professional APCs has been reported, and several studies showed that DCs present exogenous antigens to MHC class I molecules [122, 123]. Kim *et al*. showed that antigen-specific T-cell responses were enhanced by using recombinant protein antigen fused with LL-37 in vivo, compared with the use of antigen alone [124]. Especially, it was confirmed that effector CD8^+^ T cells were generated through cross-presentation of antigens. HDPs can rapidly permeabilize cellular membranes, but most HDPs show relatively little toxicity to eukaryotic cells. HDPs, such as LL-37 and BDs, have the potential to induce cross-presentation of antigens by facilitating uptake of macromolecules, such as nucleic acids and proteins, through temporary membrane disruption or transition pore opening without cell lysis [125, 126]. Therefore, HDP-mediated cell permeabilization may affect the presentation of antigens by APCs to immune cells, resulting in the alteration of antigen-specific cellular immune responses. These studies suggest that application of HDPs to vaccine platforms may overcome the limitations of subunit vaccines by inducing cellular immune responses through cross-presentation of antigens. However, many questions remain regarding the mechanisms by which HDPs act on adaptive immunity, including the possibility of antigen cross-presentation. Identification of specific cellular responses and associated signaling pathways that respond to HDPs will provide further understanding of the roles of HDPs in linking innate and adaptive immunity.

## Conclusions

We reviewed the biological functions of HDPs, including the direct antimicrobial and indirect immunomodulatory activities of these peptides. HDPs are primary components of innate host defenses and represent not only a link between innate and adaptive immunity, but may also have other bioactivities. Based on the properties discussed above, various HDPs and their derivatives are being studied extensively, including their use as antimicrobials and anti-inflammatory agents, as well as their application in cancer prevention and treatment, and wound healing. In particular, HDPs are attracting attention as alternatives to commonly used antibiotics and artificial food preservatives. Further research is required to assess the full potential of HDPs as novel immune adjuvants and immunotherapeutic agents. Overall, HDPs have good prospects for development in the pharmaceutical and food industries, and in various areas of healthcare.

## Figures and Tables

**Table 1:** Actions of Antiviral HDPs.

HDPs	Viruses	Proposed mechanisms	References
Cathelicidins			
LL-37	Dengue virus Hepatitis C virus Human immunodeficiency virus Human rhinovirus Herpes simplex virus Influenza virus Respiratory syncytial virus Vaccinia virus Venezuelan equine encephalitis virus Zika virus	Direct interaction with viruses Triggering of innate immune response Increasing IFN pathway Decreasing proinflammatory cytokine production Modulating neutrophil response	127–136
α-Defensins			
hNP-1 hNP-1, -2, and -3 hNP-4 HD-5	Human immunodeficiency virus Adenovirus Herpes simplex virus Influenza virus Papillomavirus Vesicular stomatitis virus Human immunodeficiency virus Herpes simplex virus Human immunodeficiency virus Herpes simplex virus Papillomavirus	Direct interaction with viruses Direct binding to cell receptors blocking entry Reduction of cell trafficking Releasing inhibition of viral components from endosomes Modulating innate immunity Decreasing proinflammatory cytokine production Modulating pDC, monocyte, and neutrophil responses	137 63, 138–141 139, 142 139, 143, 144
β-Defensins			
hBD-1 hBD-2 hBD-3	Herpes simplex virus Influenza virus Sendai virus Adenovirus Human immunodeficiency virus Respiratory syncytial virus Rhinovirus Human immunodeficiency virus Herpes simplex virus Influenza virus Vaccinia virus		59 145–148 139, 140, 146, 149
